# Supplementary material for: Relationship between knowledge, attitude, and practice of COVID-19 precautionary measures and the frequency of infection among medical students at an Egyptian University
Source: PLoS One. 2022 Sep 19;17(9):e0274473. doi: 10.1371/journal.pone.0274473 (PMC9484682; doi:10.1371/journal.pone.0274473)
Supplement: Attachment — Submitted filename: Response to Reviewers.docx [file pone.0274473.s002.docx]

**Rebuttal letter**

We thank the editor and the respectable reviewers for their generous comments on our manuscript “Relationship between Knowledge, Attitude, and Practice of COVID-19 precautionary measures and the Frequency of infection among medical students at an Egyptian University”. Below is our response to each point raised by the academic editor and reviewers. We have edited the manuscript to address their concerns. We hope that we satisfyingly addressed them; and that the manuscript will be now suited for publication.

Sincerely, On behalf of authors,

Ghada Wassif

Associate Prof. of Public Health, ASU

**Academic editor:**

**1- It is original paper but new idea about the relation between vaccination history and reinfection by COVID-19 among medical students, also** **if reinfection coincides with the appearance of viral mutations time is not presented.**

We truly appreciate rating the originality of our paper as very good, yet the academic editor raised two new objectives that were not actually included in our research objectives, and which would be considered as new ideas for future work. **The first one** is the relation between vaccination history and re-infection by COVID-19, which was not inquired about in our study as at the time of study conduction the vaccination status among medical students representing part of the adolescents population in Egypt was low. Adolescents in Egypt received the vaccination only when the Egyptian government started preventing students from entering universities without a valid coronavirus vaccination certificate. In addition to that, this information is also supported by an Egyptian study carried out by Saied et al., 2021 who mentioned that although the majority of medical students (90.5%) perceived the importance of the COVID-19 vaccine, 46% had vaccination hesitancy. So were considering in our study the precautionary measures taken by medical students such as wearing facemasks, Handwashing, Social distancing, etc…

An interesting table reflecting the relationship between practicing precautionary measures among medical students and the frequency of infection was added to the results section **Page 13-15** Which shows that there was a statistically significant relationship between the frequency of infection in medical students and their conformance with COVID-19 precautionary measures such as wearing masks and practicing social distancing in lecture halls, and frequently washing their hands.

This information is available at:

<https://www.arabnews.com/node/1941241/middle-east>

[Vaccine hesitancy: Beliefs and barriers associated with COVID‐19 vaccination among Egyptian medical students (wiley.com)](https://onlinelibrary.wiley.com/doi/epdf/10.1002/jmv.26910)

**The second one** is if reinfection coincides with the appearance of viral mutations time or not; which was not also actually inquired about in our study as were asking medical students about the frequency of infections and not about the exact time of each infection as this also might be subjected to recall bias.

**2- The language quality of your manuscript is not sufficient to send it out for external review. Please contact a professional editing service, or at least a native-speaking colleague before you resubmit an improved version. “Need English editing”.**

The article had been submitted for English language editing by Enago, the editing brand of Crimson Interactive Inc. under Copyediting/Language editing and they provided us with a certificate proving; that is made available with the submitted files that the manuscript has been edited for English language, grammar, punctuation, and spelling. They also checked the adherence to journal guidelines and made required changes.

**3- The research question is not well demarcated**

The following sentence had been added at the end of the introduction clarifying the research question and study objective “Page 4 Line 84-91”.

“The present study intends to explore if a relationship exists between the KAP of medical students related to COVID-19 precautionary measures and the frequency of infection in an endeavor to provide evidence that the conformance of medical students to COVID-19 precautionary measures can substantially reduce the incidence of infection. This objective is in line with the notion that infection may occur once due to inevitable contact with an infected family member. However, frequent infection could be prevented by practicing precautionary measures, which could decrease the burden of COVID-19 on the health care system and reduce the cost of treatment. In addition, mortalities associated with COVID-19 could be reduced effectively”.

**4- The increased infection rate among students who attend clinical round is not well analyzed; is reinfection related to their round in the chest department or in different departments without sufficient precautions or due to overcrowding of patients without quarantine.**

It is very difficult to relate infection to clinical round attendance as during COVID-19 infection other factors would be considered which are overcrowding of students in lecture halls whether in the university or the hospitals without practicing social distancing or wearing face masks. Students may also contact infection if they don’t adequately practice hand washing or sterilization of hands with alcohol.

In addition to that, the present study aimed to relate the increased infection rate to the inadequate practice of precautionary measures toward COVID-19 regardless of the source of infection (whether students had contracted the infection from the hospital or overcrowding of students in lecture halls).

**5- No clear pointing on adequate health education tools or precautionary measures taken by the author to change the attitude**

This is a great point to be considered by the authors for future studies to increase students’ conformance with precautionary measures and vaccine acceptance as well in an attempt to decrease future infections with COVID-19; However, for the present study it is rather an Analytical Non-Interventional Comparative cross-sectional study; that attempts to prove that conformance with precautionary measures toward COVID-19 would substantially result in lower infection rates among medical students.

**6- Inapparent key points in the paper make it unique than paper done in Jordon.**

The present study cited three papers that were carried out in Jordan; The first one was carried out by ***Khasawneh et al., 2020*** was totally different from our paper. ***Khasawneh et al., 2020*** is a descriptive study that represented the first evaluation of the COVID-19 outbreak's knowledge, attitudes, and precautionary measures amongst medical students in Jordan. However, our study is a comparative analytical cross-sectional study that explores the relationship between students’ knowledge, attitude, Practice of COVID-19 precautionary measures, and the frequency of infection among medical students at Ain Shams University, Cairo, Egypt.

To our knowledge, our study is the first one to explore such a relation in an attempt to highlight the importance of conformance of precautionary measures toward COVID-19 and the reduction of infection in medical students

Moreover, ***Khasawneh et al., 2020*** carried out his study among healthcare providers not medical students, he also used a different study tool from ours.

The second study in Jordan was carried out by ***Hani et al., 2021*** which is a cross-sectional study, designed to (1) determine the incidence of COVID-19 in clinical and preclinical medical students (2) determine if there is a difference in infection rates between clinical and preclinical students.

Hani et al., 2021 differed from our study in that they didn’t assess the knowledge, attitude of medical students towards COVID-19, however, they studied only three precautionary measures not in details as our study. They only related the precautionary measures to the infection incidence rates; but our was totally different as well; as we are minded more with the relationship between knowledge, attitude and practice of precautionary measures and being infected with COVID-19 more than once (i.e., the frequency of infection not just the infection that occurred once or for the first time).

The third study was carried out by Mustafa et al., 2020 and it was far different from ours as it is a descriptive study aimed to assess knowledge, attitude, and infection control measures related to the coronavirus disease 2019 (COVID-19) pandemic outbreak among Jordanian health care students.

**7- Discussion is long, in methodology (information about Ain shams without mention the time of the study.**

The discussion had been shorted and changes are marked in track changes, Renumbering of references had been carried out.

Study Time was added to the Methodology section on Page 4 Lines 98-101 as follows “The participants were undergraduate medical students in their first to sixth academic years who agreed to participate in the study. Students were recruited across three months, that is, from June to August 2021 via the snowball sampling technique”

**8- Data availability statement not in the manuscript**

The Following statement regarding data availability is added on Page 22 Lines 367-369 as follows

Data Availability Statement

“The raw data supporting the conclusions of this article will be made available by the authors without undue reservation.”

**Additional Editor Comments:**

**Reviewer #1:**

**1- The paper needs English editing to be more interesting to the readers.**

The article had been submitted for English language editing by Enago, the editing brand of Crimson Interactive Inc. under Copyediting/Language editing and they provided us with a certificate proving; that is made available with the submitted files that the manuscript has been edited for English language, grammar, punctuation, and spelling. They also checked the adherence to journal guidelines and made required changes.

**2- More focus on the aim, the connection between reinfection with COVID-19 among medical students and infection control procedures in the hospital.**

The following sentence had been added at the end of the introduction clarifying the research question and study objective “Page 4 Line 84-91”.

“The present study intends to explore if a relationship exists between the KAP of medical students related to COVID-19 precautionary measures and the frequency of infection in an endeavor to provide evidence that the conformance of medical students to COVID-19 precautionary measures can substantially reduce the incidence of infection. This objective is in line with the notion that infection may occur once due to inevitable contact with an infected family member. However, frequent infection could be prevented by practicing precautionary measures, which could decrease the burden of COVID-19 on the health care system and reduce the cost of treatment. In addition, mortalities associated with COVID-19 could be reduced effectively”.

A table displaying the relationship between frequency of infection in medical students and Practice of COVID-19 Precautionary measures had been added on Page 13-15.

**Reviewer #2:**

**1- The manuscript should be sent for English proofreading.**

The article had been submitted for English language editing by Enago, the editing brand of Crimson Interactive Inc. under Copyediting/Language editing and they provided us with a certificate proving; that is made available with the submitted files that the manuscript has been edited for English language, grammar, punctuation, and spelling. They also checked the adherence to journal guidelines and made required changes.

**2- Referencing style must be followed as per the journal’s guidelines.**

References had been revised and updated per the journal’s guidelines.

**3-Authors must strengthen the rational/problem statement of the study objectives specific to the importance of KAP precautionary measures and medical students, of course, previous studies won’t be available as COVID19 is a new phenomenon, but authors can justify it in other ways.**

Rational/study justification had been added at the end of the introduction section and the modifications had been marked with track changes (Page 4 Line 84-91).

Our study rationale is that infection may occur once due to inevitable contact with an infected family member. However, the frequent infection could be prevented by practicing precautionary measures, which could decrease the burden of COVID-19 on the health care system and reduce the cost of treatment. In addition, mortalities associated with COVID-19 could be reduced effectively”.

**4-Objectives must be added clearly in the introduction section.**

The following sentence had been added at the end of the introduction clarifying the research question and study objective “Page 4 Line 84-91”.

“The present study intends to explore if a relationship exists between the KAP of medical students related to COVID-19 precautionary measures and the frequency of infection in an endeavor to provide evidence that the conformance of medical students to COVID-19 precautionary measures can substantially reduce the incidence of infection.

**5-How was the validity and reliability of the questionnaire/study tool established?**

The questionnaire was designed by ***Erfani et al., 2020*** based on WHO training material for the detection, prevention, response, and control of COVID-19. Validity and reliability were tested by questionnaire authors and details had been added to the study tools in the methodology section on Page 5, Lines 113-121.

**6-Ethical approval should be written in a more scientific way excluding minor details like the dean’s and vice dean’s approval.**

The ethical approval statement was modified, and minor details were deleted with track changes.

**7-I wonder, how qualitative data can be presented in numbers?**

The word had been corrected on Page 6 Line 158; Categorical data as nominal and ordinal variables are presented in numbers and their related frequencies.

**8-Discussion must include the generalizability (external validity) of the study results**

A statement clarifying the generalizability (external validity) of study results had been added at the end of the discussion in addition to the study limitations on Page 21, lines 345-351.
